# From Nature to Innovation: Advances in Nanocellulose Extraction and Its Multifunctional Applications

**DOI:** 10.3390/molecules30132670

**Published:** 2025-06-20

**Authors:** A. M. P. Hansini, G. D. C. P. Galpaya, M. D. K. M. Gunasena, P. M. Abeysundara, V. Kirthika, L. Bhagya, H. D. C. N. Gunawardana, K. R. Koswattage

**Affiliations:** 1 Center for Nanodevice Fabrication and Characterization (CNFC), Faculty of Technology, Sabaragamuwa University of Sri Lanka, Belihuloya 70140, Sri Lanka; prathibhaandramana@gmail.com (A.M.P.H.); chanakagalpaya@gmail.com (G.D.C.P.G.); kasundi@tech.sab.ac.lk (M.D.K.M.G.); rvkeerthikarv@gmail.com (V.K.); lakshanibhagya96@gmail.com (L.B.); niroshan@tech.sab.ac.lk (H.D.C.N.G.); 2 Department of Biosystems Technology, Faculty of Technology, Sabaragamuwa University of Sri Lanka, Belihuloya 70140, Sri Lanka; maduabesundara123@gmail.com; 3 Faculty of Graduate Studies, Sabaragamuwa University of Sri Lanka, Belihuloya 70140, Sri Lanka

**Keywords:** nanocellulose, extraction methods, cellulose, applications, nanofibrils, acid hydrolysis, chemical treatments

## Abstract

Nanocellulose obtained from renewable and abundant biomass has garnered significant attention as a sustainable material with exceptional properties and diverse applications. This review explores the key aspects of nanocellulose, focusing on its extraction methods, applications, and future prospects. The synthesis of nanocellulose involves mechanical, chemical, and biological techniques, each uniquely modifying the cellulose structure to isolate cellulose nanocrystals (CNCs), cellulose nanofibers (CNFs), or bacterial nanocellulose (BNC). These methods provide tailored characteristics, enabling applications across a wide range of industries. Nanocellulose’s remarkable properties, including high mechanical strength, large surface area, thermal stability, and biodegradability, have propelled its use in packaging, electronics, biomedicine, and environmental remediation. It has shown immense potential in enhancing the mechanical performance of composites, improving water purification systems, and serving as a scaffold for tissue engineering and drug delivery. However, challenges related to large-scale production, functionalization, regulatory frameworks, and safety concerns persist, necessitating further research and innovation. This review emphasizes the need for sustainable production strategies and advanced functionalization techniques to harness nanocellulose’s full potential. As an eco-friendly, high-performance material, nanocellulose presents a promising avenue for addressing global sustainability challenges while offering transformative solutions for various industries.

## 1. Introduction

For the past few years, special attention has been focused on the development of new composite materials that are cost-effective and exhibit strong performance characteristics, with a particular focus on environmentally friendly materials. There are several comprehensive reviews that explore the progress in composite materials derived from renewable resources, highlighting biodegradable polymers and their structural properties, morphology, and processing techniques [1,2]. Based on that fact, biodegradable polymers have gained significant attention over the past few decades due to their potential applications in environmentally conscious fields such as packaging and agriculture, as well as in healthcare, including regenerative therapies and controlled drug delivery [3,4]. The rise in research related to biopolymers is largely driven by their contribution to reducing carbon emissions and decreasing reliance on fossil fuels, leading to a global surge in demand for polymers sourced from renewable resources. Among these polymers, cellulose and starch have gained attention for composite material applications. In this article, the main focus will be on the properties and emerging applications of cellulose, which have been discovered over the last few years. This review provides a thorough and integrated perspective on nanocellulose based on nearly all of the extraction techniques reported under three categories: mechanical, chemical, and biological. Furthermore, readers will have a detailed, all-in-one overview of extraction strategies, as well as the major application areas of nanocellulose with uses in various fields. This review continues by discussing challenges and provides future directions to improve nanocellulose preparation and use. This work incorporates breadth, depth, and clarity and will be a useful and practical resource for researchers and industries alike. 

### 1.1. Cellulose and Nanocellulose

**Figure 1:**
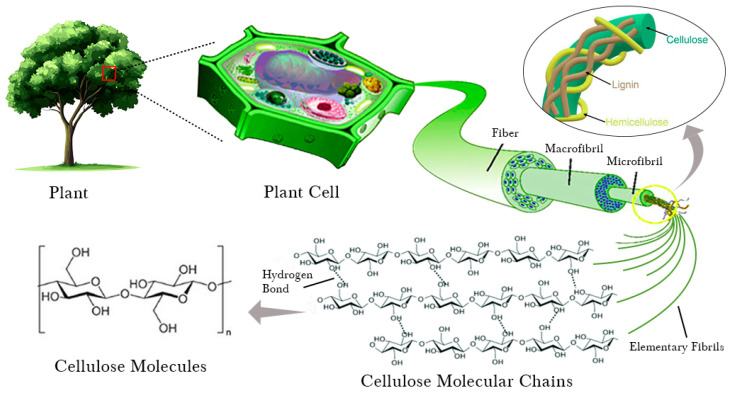
Illustration of the hierarchical structure of cellulose fibers.

Cellulose, the most abundant polymer on Earth, consists of linear macromolecules made up of β-1,4-linked D-glucopyranose units. In plants, cellulose exists in the form of microfibrils (2–20 nm in diameter and 100–40,000 nm in length), which provide structural strength to the cell walls, as further illustrated by Figure 1 [5]. Thus, it plays a significant role as a primary structural material in plants and various living organisms, helping them to maintain their form. Although individual cellulose strands are neither more hydrophobic nor less hydrophilic than other soluble polysaccharides such as amylose, cellulose’s extensive intra- and intermolecular hydrogen bonding leads to its tendency to crystallize, rendering it insoluble in typical aqueous solutions, which is applicable for various applications [5].

When cellulose fibers undergo mechanical shear or controlled acid hydrolysis, they produce elongated, fibrillar rod-like particles with nanoscale widths and lengths ranging from 50 nanometers to several micrometers [6]. Nanocellulose, which can either be extracted from plant sources or produced biochemically in the lab, retains many of cellulose’s desirable traits, such as low density, nontoxicity, and excellent biodegradability. However, nanocellulose also exhibits unique properties, including high mechanical strength, reinforcing potential, and tunable self-assembly behavior in aqueous environments, resulting from its distinct shape, size, surface chemistry, and high crystallinity [6]. In recent decades, nanotechnology involving cellulosic substrates has gained significant attention. Sustainable development requires the creation of products that are renewable, environmentally friendly, and non-petroleum-based, pose minimal health and safety risks, and remain economically viable. Nanocellulose, with a Young’s modulus ranging from 20 to 50 GPa and a surface area of several hundred square meters per gram [7], offers many promising properties and applications. As an excellent alternative to petroleum, biomass is now recognized as a valuable resource for biofuels and chemicals.

### 1.2. Types of Nanocellulose

There are three primary types of cellulose: (1) cellulose nanofibers (CNFs), (2) cellulose nanocrystals (CNCs), and (3) microbial or bacterial nanocellulose (BNC). Cellulose nanofibers and cellulose nanocrystals are also known as nano-fibrillated cellulose (NFC) and micro-fibrillated cellulose (MFC) [6,8]. Even though the chemical and physical characteristics based on the origin and purification procedures may differ, the molecular backbone of cellulose is similar to all three types of nanocellulose [9]. *Acetobacter xylinum* (*Glucanacetobacter xylinus*), a Gram-negative bacterium, is considered the main source of BNC or bacterial cellulose (BC). In addition, microbial sources such as *Alcaligenes, Sarcina*, *Acetobacter*, *Pseudomonas*, *Rhizobium*, and *Archromobacter* are capable of producing nanocellulose [10].

#### 1.2.1. Cellulose Nanofibers (CNFs)

CNFs primarily consist of cellulose fibrils embedded within a reinforcing matrix, affording superlative rigidity, tensile strength, and flexural properties [11]. This makes CNFs a promising tool for greener material development with enhanced properties, contributing to the sustainable management of environmentally friendly materials. Regarding the industrial viability of a CNF, it must demonstrate both qualitative and quantitative performance at each step of its lifecycle. Due to its unique nanoscale morphology, remarkable mechanical strength, high crystallinity, and large specific surface area, nanocellulose has become a distinctive natural material, attracting increasing interest from both academia and industry since nanotechnology made its development possible [12]. According to [7], nonwoven CNF nanostructures with a consistent width of 15 nm, prepared by a simple mechanical process, exhibit interconnected pore structures with an extensive surface-to-volume ratio, suitable for a range of applications such as filters, membranes, and tissue scaffolds.

#### 1.2.2. Cellulose Nanocrystals (CNCs)

CNCs, typically isolated utilizing acid hydrolysis, have rod-like, cylindrical nanoparticles characterized by their relatively rigid structure. The usual dimensions range between 4–70 nm in width, with lengths between 100–6000 nm, while the crystallinity index is 54–88% [13]. Over the past two decades, these nanomaterials have been described under various names, including nanocrystalline cellulose, nanoballs, cellulose crystallites, cellulose nanowires, nanorods, cellulose nanowhiskers, and cellulose whiskers [14,15]. In recent years, however, the terminology has gradually converged, with “cellulose whiskers”, “cellulose nanowhiskers”, and, more recently, “cellulose nanocrystals” and “nanocrystalline cellulose” becoming the most widely accepted terms [16].

#### 1.2.3. Bacterial Nanocellulose (BNC)

BNC and its composites are increasingly utilized in temporary wound healing systems [17], such as tissue regeneration and drug delivery [18]. The first significant use of BNC in biomedicine was the development of BioFill in 1990, a wound dressing designed to treat severe burns, wounds, skin grafts, and chronic ulcers [19]. Another milestone was achieved in 2001 with the creation of BNC-based artificial blood vessels for microsurgical procedures [20]. However, the practical use of BNC largely hinges on its production efficiency. High capital investment and costly manufacturing processes present significant challenges to the commercialization of BNC at an affordable price [21].

## 2. Extraction Methods

The methods for the extraction of the nanocellulose can be grouped under categories such as mechanical, chemical, and biological approaches. These methods can also be combined in ways that optimize quality, yield, and environmental sustainability. The type of extraction method chosen is based on the outcome of obtaining nanocellulose with desired properties, fields, or applications for these products and the nature of the origin of the cellulose.

### 2.1. Mechanical Methods

Mechanical approaches cause physical stresses to cellulose fibers, causing them to disperse in the nanoscale, resulting primarily in CNFs. These processes use high pressure, shearing, and ultrasonic energy to separate the tiny cellulose hydrogen-bonded structure into nanosized scales. The application of high mechanical forces breaks apart these cellulose microfibrils interconnected by Van der Waals and hydrogen bonds to produce smaller fibrils. Although the crystalline regions of cellulose remain intact, its amorphous portions will be disrupted, causing an increase in fibril surface area and compliance [22]. These are chemical-waste-free and scalable. Nevertheless, they are energy-demanding, and great amounts of chemicals or enzymes will have to be loaded as a pretreatment to lower power consumption [23].

#### 2.1.1. High-Pressure Homogenization (HPH)

HPH is used to shear cellulose fibers in multiple passes under a high pressure of 50–2000 MPa and causes fibrillation due to the forced flow through constricted areas [24]. These forces break up the hydrogen bonds that result in the opening of microfibrils, leading to nanofibrils with a diameter between 20–100 nm [25]. It has been commonly used for the production of CNFs at the industrial scale, even though it is relatively more energy-intensive. HPH may constitute an effective approach for fiber refinement as it is considered a highly effective, simple, and solvent-free approach [26]. HPH for the production of nanocellulose from wood pulp was first applied in 1983 [27]. Researchers have used different raw materials in HPH. Since then, research has reported 10–15 cycles of HPH at 30 MPa for 3 days to isolate nano-fibrillated cellulose from bleached sugar beet [28], while some bleached cellulose residue from the skin of prickly pears could be used to extract nano-fibrillated cellulose with a diameter of around 2–5 nm by blending up to 15 cycles at 50 MPa at a temperature below 95 °C [29].

One significant issue regarding HPH is the clogging problem due to its very small orifice size. To avoid this shortcoming, the fibers are reduced in size prior to HPH. For this purpose, different mechanical treatments are applied before the HPH process. This is done by applying HPH on fibers, which researchers have performed on kenaf bast fibers [30], cores [31], and stems [32]. They used refining and cryocrushing pretreatments for kenaf bast fibers and obtained nanofibers with an average diameter of 10–90 nm. The implemented grinding pretreatment for the kenaf cores and stems resulted in nanocellulose with widths of 20–25 nm and 15–80 nm, respectively. Refining may also serve as a pretreatment step prior to HPH. It consists of a disk refiner, which contains a diluted fiber suspension flowing in a gap that is in between the stator and rotor disks.

#### 2.1.2. Grinding

Mechanical friction involves the grinding of cellulose fibers using a disk mill to produce nanoscale fibrils and peeling the fibrils layer by layer. This breaks the amorphous areas, leaving a semi-crystalline structure behind. It is typically combined with chemical or enzymatic pretreatments to increase efficiency [33].

On bleached eucalyptus pulp with nanocellulose, a commercial stone grinder was employed to apply a grinder to produce nanocellulose [34], where the range of electrical energy input was between 5 and 30 kWh/kg. The authors established a correlation between the energy consumed and the fibrillation time as a function of crystallinity and the degree of polymerization (DP) by applying a range of energy inputs. The results showed that after 11 h, the DP (850–550) and crystallinity index (62–40) decreased as the energy input increased from 5 to 30 Wh/kg. The authors mentioned that the evaporation of water and the solid mass fraction due to the heat of friction in the fibrillation process increased from 2 to 3.2% after 11 h, which caused an increase in the specific fibrillation energy. Another study used a high-shear grinder and homogenizer for bleached rice straw and bagasse pulps to produce nanofibers with passages of 30 and 10 times, respectively. They discovered that refining was the dominant mechanism for nanocellulose isolation, while HPH resulted in smaller and more uniform nanofibers. Conversely, using a high-shear grinder alone was insufficient to finish the fibrillation process in HPH. In addition, the findings showed that these two steps did not have a harmful influence on the polymerization degree of these fibers [34].

#### 2.1.3. Cryocrushing

Brittle fractures can be created by freezing bulk cellulose in liquid nitrogen, followed by crushing between two surfaces, resulting in nanofibrils. Cryocrushing is particularly useful in obtaining fibrils with well-defined dimensions [35]. Applying high-impact forces on the frozen cellulosic fibers causes the cell walls to rupture by exerting pressure from the ice crystals and releasing nanofibers [36]. Nanofibers from soybean stock were generated by cryocrushing and high-pressure defibrillation processes [37]. The average diameter of the soybean stock nanofibers was observed to be 50–100 nm from transmission electron microscope (TEM) results, and their dispersion in an acrylic oligomer emulsion was better than in water. The X-ray diffraction (XRD) analysis showed that its crystallinity was approximately 48%. It is not a widely used method, but it still allows a very good degree of control over the nanocellulose morphology that forms.

#### 2.1.4. Ultrasonication

High-frequency sound waves of around 20 kHz form cavitation forces to break cellulose fibers into nanoscale structures. As the bubbles burst, they create extreme shear forces that shred fibrils into fragments on a nanoscale. Ultrasonication has been reported to reduce agglomeration and enhance dispersion [38]. High-intensity ultrasonication treatment and oscillating forces are the subject of various studies devoted to isolating nanofibers from cellulosic sources. Ultrasonication is commonly used to disaggregate nanocellulose in combination with chemical pretreatments [39,40].

Wang and Cheng studied the influence of temperature, concentration, power, size, time, and distance from the probe tip on some cellulose fibers on the degree of fibrillation using ultrasonication. They found that higher power and temperature led to better fibrillation, whereas longer fibers decreased fibrillation. However, both concentration and a larger distance between the probe and the beaker were unfavorable for fibrillation [41]. When ultrasound-TEMPO (2,2,6,6-tetramethylpiperidine-1-oxyl) oxidization was applied, the yield of nanocellulose production was 71% because of the cavitating bubbles in ultrasonication. It was concluded that mechanical treatment using a blender (yield of 90% in 40 min) and an ultrasound probe (yield of 100% in 25 min) with a higher ultrasonication intensity was more efficient in the production of nanocellulose than an ultrasound bath (yield of 50% in 60 min) [42]. Chen and colleagues in 2011 showed that the crystallinity increased to over 60%, whereas the degradation temperature was more than 330 °C when nanofibers were extracted from bamboo, wood, and wheat straw using a 30 min ultrasonic treatment with power at 1000 W and frequency in the range of 20–25 kHz [43].

### 2.2. Chemical Methods

Chemical treatments frequently precede mechanical methods used to get rid of hemicellulose and lignin from cellulose, causing nanocellulose isolation suitable for the formation of CNCs possessing a high crystallinity. Chemical treatments are directed toward the amorphous regions of cellulose, leaving the crystalline domains behind. This includes the acid hydrolysis of glycosidic linkages, which degrades amorphous regions preferentially to yield highly crystalline CNCs [44]. Chemically derived nanocellulose provides an opportunity for a high degree of control on the properties at a lower cost than mechanical methods but can produce substantial chemical waste and associated neutralization following treatments [45].

#### 2.2.1. Acid Hydrolysis

Acid hydrolysis is an example of a process for chemically extracting nanocellulose from cellulosic materials. Since cellulose chains contain ordered and disordered areas, the disordered parts are easily hydrolyzed by acid and the ordered portions remain as residue. For this purpose, various mineral acids can be applied, including sulfuric, hydrochloric, phosphoric, maleic, nitric, and formic acids [46,47,48,49]. Acid mixtures (hydrochloric and organic acids such as acetic or butyric acids) have also been studied [50].

Strong acids such as sulfuric acid may catalyze the hydrolysis of glycoside bonds in cellulose. This can not only effectively isolate nanocrystalline cellulose but also cause the presence of the nanocellulose to be dispersed as a stable colloid system by the esterification of the hydroxyl group by sulfate ions [51]. Consequently, this partial degradation of amorphous regions enhances the crystallinity index with different CNC dimensions based on hydrolysis conditions [52]. Sulfate ester groups (from sulfuric acid) have a negative surface charge and, correspondingly, the stability of an aqueous medium through this static potential is increased [53]. Reaction time, temperature, and acid concentration are the main controlling factors that have an effect on the properties of the obtained nanocellulose [54]. However, a significant disadvantage of acid hydrolysis is represented by the acid wastewater resulting from the wash, which adjusts the pH value of the nanocellulose suspension.

The influence of hydrolysis conditions on the yield, morphology, and properties of CNCs has been studied recently [55]. Generally, a higher acid concentration, longer reaction time, and higher temperature could intensify the surface charge and narrow the size but would decrease the yield and lower the crystallinity and thermal stability of nanocellulose. For instance, the CNC yield is about 30% after applying 63.5 wt% (sulfuric acid) SA for cellulose hydrolysis, but when 65 wt% SA was used for cellulose hydrolysis, the CNC yield decreased to ≤20%. The CNC yield reached its highest value of 65–70% when the SA concentration was reduced to 60 wt% [56].

Recently, Juan Guo and colleagues showed the effect of ultrasound on cellulose hydrolysis [57], where the ultrasonication treatment, simultaneous with the hydrolysis reaction, increased CNC yields with short hydrolysis times (45 min). On the other hand, the widths and thicknesses of CNCs were reduced by further acid hydrolysis in ultrasonication due to the delamination and disorder of the cellulose crystalline structures that emerged from the partial dissociation of cellulose hydrogen bond networks presented in CNCs during ultrasound treatment with long hydrolysis times.

#### 2.2.2. Alkali Treatment and Bleaching

Alkalization removes hemicellulose and lignin, leaving pure cellulose for the next processing steps. Afterward, the solid products were washed with distilled water to a neutral pH and finally dried in an oven at 50 °C. The fiber products obtained from this treatment were primarily cellulose, and other non-cellulosic substances were eliminated. Sodium hydroxide is a frequently used agent in pretreatments [58]. Hemicellulose is removed by sodium hydroxide, whereas lignin can be eliminated by bleaching with oxidizing agents (e.g., hydrogen peroxide). First, these treatments break down the cellulosic swelling barriers on cellulose fibrils, which then become accessible for fibrillation. Oxidants, such as sodium chloride or hydrogen peroxide, remove the remaining lignin and provide white cellulose with a high potential for fibrillation. Bleaching increases the whiteness and fibrillation degree of fibers [36,59].

Many groups in recent times used these biomass pretreatment methods for the removal of non-cellulosic materials in agricultural wastes. For instance, Nurain Johar and his team modified rice husks with an alkaline, followed by bleaching. They observed that the cellulose content was enhanced from 31 wt% (in untreated rice husks) to 96 wt% after alkaline and bleaching pretreatments. Additionally, the products did not contain lignin, hemicellulose, or any other non-cellulosic materials [60]. Additional agricultural wastes, including oil palm empty fruit bunch, sugarcane bagasse, pineapple leaf, apple stem, coir fiber, mulberry bark, soybean hulls, and cotton linters, have also been pretreated prior to the extraction of nanocellulose [59,61,62,63].

In the bleaching process, the pulp can be bleached to get rid of residual lignin and other impurities without altering the cellulose’s crystallinity or polymorphism. The resulting white cellulose has enhanced aging stability after the bleaching process. Bleaching agents that can be used include hydrogen peroxide, oxygen, ozone, peracetic acid, sodium chlorite, chlorine, and chlorine dioxide. Oxygen and chlorine dioxide are the most frequently used bleaching agents. Hubbe and colleagues have provided additional information about bleaching processes [64].

The selective oxidation of primary hydroxyl groups in cellulose to carboxyl groups is crucial and this process is mediated by the TEMPO catalyst. The obtained CNF possesses higher dispersibility and surface functionality; hence, is applicable to diverse fields [65]. Applications that are best suited for this method would be those requiring high levels of transparency and flexibility. The selective oxidation of C6 primary hydroxyl groups to carboxyl groups by TEMPO provides anionic functionalities in cellulose, giving the hydrophobic nanocellulose surface. The modification of exposed C6 alcoholic group-based functions of the C_6_H_12_O_6_ (glucose) units to carboxylic acid (–COOH), as well as stable nitroxyl radicals, transformed the hydroxyl (–OH) groups into aldehydes, which were then further oxidized to carboxylic acids [66,67]. Table 1 illustrates several sources of nanocellulose and their resultant nanocellulose types and dimensions.

### 2.3. Biological Methods

The biological methods use enzymes or microorganisms to degrade the cellulose or simply produce BNC. From an environmental standpoint, these methods are environmentally friendly but are less efficient for industrial-scale production. The amorphous regions of cellulose can be slowly degraded by biological processes that lead to individual fibrils or new highly crystalline material created directly from glucose precursors [77].

#### 2.3.1. Enzymatic Hydrolysis

Cellulose enzymes act on specific glycosidic bonds, helping to break down cellulose fibers into the nanofibrils scale. Although this method promotes higher environmental sustainability, its performance is lower than the one set by conventional mechanical and chemical approaches [78]. The enzymatic pretreatment is a green alternative to chemical pretreatments. The lignin and hemicelluloses can be bio-transformed by special enzymes also known as ligninases, xylanases, etc., which degrade lignin and hemicellulose but preserve cellulose. In contrast, cellulolytic enzymes, that is, cellulases, assist in the hydrolysis of cellulosic fibers [79].

**Figure 2:**
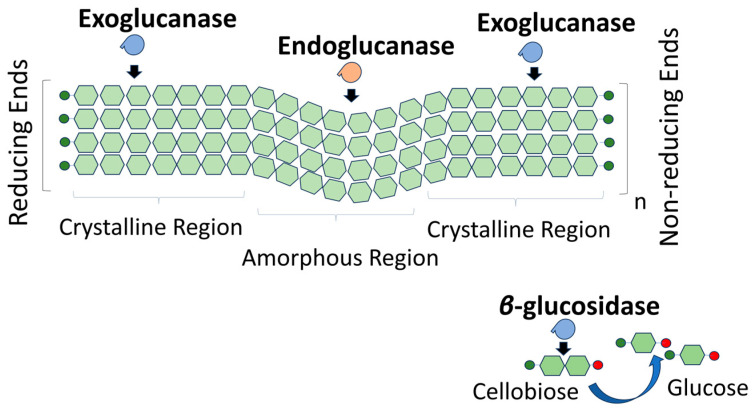
Classification of cellulases based on their enzymatic activity on cellulose fiber.

Based on their enzymatic action, cellulases can be classified into three categories: (i) endoglucanases or β-1,4-endoglucanases (namely A- and B-type cellulases), which randomly hydrolyze accessible β-1,4-glucosidic linkages occurring in noncrystalline regions of cellulose to produce damaged fibers with new chain ends; (ii) exoglucanases (cellobiohydrolases), which are also referred to C- and D-type cellulases, that target the chain ends to release soluble cellobiose as the predominant product; and (iii) β-glucosidases that hydrolyze cellobiose, giving rise to glucose. Figure 2 depicts the action of each enzyme on crystalline and amorphous regions of the cellulose fiber. According to the widely accepted mechanism of cellulose hydrolysis, these three kinds of cellulases function synergistically [80].

CNFs have been obtained from bleached wood pulp using mild enzymatic hydrolysis with endoglucanase, followed by high-pressure disintegration. The chemical and fiber morphological analysis of the bleached wood pulp should be conducted before and after the above process operations (enzymatic hydrolysis and high-pressure disintegration). It was observed that endoglucanase pretreatment increases swelling of the pulp fibers in water and causes their disintegration, avoiding blocking or clogging of the microfluidizer. The molecular weight of the fibers was observed to be well-maintained, and successful disintegration was achieved on the fibers with the lowest enzyme concentration (0.02%). Consequently, mild enzymatic hydrolysis aids in the breakdown of pulp fibers to form nanofibers. Enzymatic pretreatment produced homogeneous CNFs with larger aspect ratios than acidic pretreatment [78,81].

Using a complex of cellulases from a fungus identified as belonging to the genus *Trichoderma* was an effective approach for cellulose with nanometric particles with a very low polydispersity. XRD, DLS, FTIR, and HPLC analyses demonstrated that the ability of cellulose to be bioconverted is dependent on which cellulolytic enzyme was used. The highest efficiency of the process of enzymatic hydrolysis was of the cellulolytic enzyme complex produced by the microscopic fungus *Trichoderma reesei*; this was confirmed by obtaining more glucose, a higher degree of crystallinity values, and a greater amount of cellulose at the nanoparticle level when compared to using a cellulolytic enzyme from *Aspergillus* sp. Notably, the ability to obtain cellulose with varying amounts of nanometric particles ranging from about 84% to 99% (depending on the type of micrometric cellulose) is possible through controlled enzymatic hydrolysis. More importantly, nanocelluloses obtained using the *Trichoderma reesei* enzyme have lower polydispersities versus materials obtained with the use of platelets from *Aspergillus* sp. [82]. There is a study that reports the activity of cellulase in biomass conversion. This provides context for the enzymatic activity used in similar applications [83].

#### 2.3.2. Microbial Fermentation

Certain bacteria, such as *Komagataeibacter xylinus*, synthesize BNC during metabolic processes. BNC is obtained in its pure (cellulose) and highly crystalline form, avoiding the necessity of extensive purification [84]. However, BNC production is expensive and has low yields, leading to the investigation of new practices, such as inexpensive substrates for lower-cost fermentations. During BNC biosynthesis, if the culture broth contains different components of organic, inorganic, or polymer materials, which can be included in the BNC membrane, this can promote the functionalization of BNC. There are primarily two synthetic strategies to prepare BNC matrix composites, namely in situ and ex situ. The in-situ method allows the addition of secondary components to the BNC culture media as the BNC synthesis process is initiated. In the ex-situ method, secondary components can be incorporated into the BN [85].

Esra Erbas Kiziltas in 2015 reported the in-situ biosynthesis of BNC, using A. xylinum 23,769 to secrete polymeric structures in the presence of different nanoparticles (CNFs, exfoliated graphite nanoplatelets [xGnPs], and nano clay [NC]). The nanoparticles showed excellent dispersion among the BC-based nanomaterials obtained with the nanoparticles embedded among the voids and microfibrils. Compared to pure BNC, it was observed that the thermal stability and residual mass of the BNC-xGnP and BNC-NC nanomaterials increased distinctly. The incorporation of CNFs into the BNC matrix did not impair the thermal stability and residual mass of the BNC matrix [85].

The selection of these extraction types is based on the intended features and applications, as well as in relation to the source material employed for obtaining nanocellulose. Though mechanical methods allow for scaling, chemical approaches can be more controlled in tuning the properties of nanocellulose. Although biological means of production are favorable to the environment, there are challenges regarding scale-up unless an efficient technological breakthrough is achieved. The combination of these approaches has the possibility to increase nanocellulose extraction efficiency while fulfilling a growing need for eco-friendly and high-performing materials. Furthermore, Table 2 depicts the key properties of nanocellulose with the corresponding influencing factors.

## 3. Applications of Nanocellulose

### 3.1. Biomedical Applications

**Figure 3:**
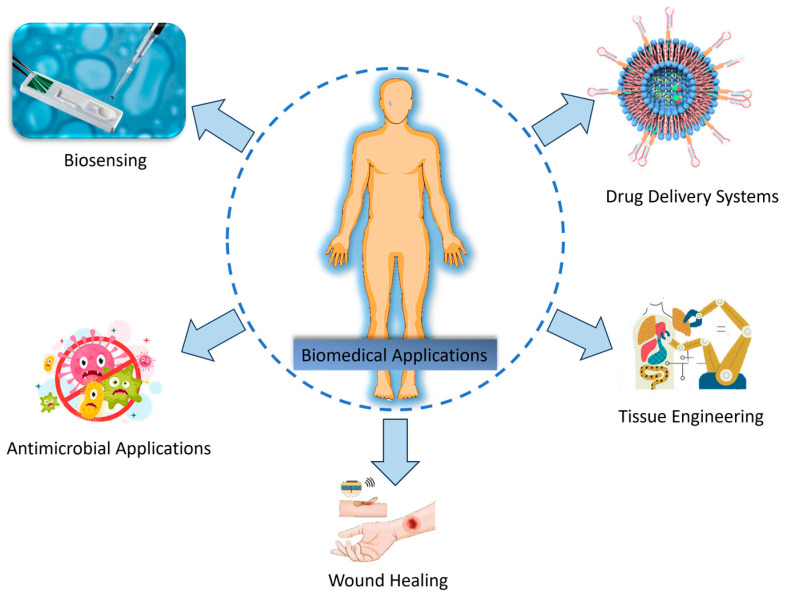
Applications of nanocellulose in the biomedical field.

Biocompatibility is one of the most attractive characteristics of nanocellulose, which renders it safe to be used in contact with human tissues and cells. Moreover, it can be broken down into its sequential decomposition products, reducing environmental pollution-related concerns about biomedical waste. Recent developments also allow the use of functionalization of nanocellulose with antimicrobial agents, growth factors, and therapeutic molecules that extend its applications in infection control, tissue regeneration enhancement, and improved drug therapies [97]. Figure 3 summarizes the key biomedical applications of nanocellulose.

#### 3.1.1. Drug Delivery Systems

In the case of hydrogels for sustained release, CNFs have been processed to form hydrogels that can trap medication and provide sustained drug release, improving its bioavailability. For example, CNF-based hydrogels could provide controlled medication release and thus improve therapeutic benefits [98]. In another aspect, CNCs have been utilized as ingredients in composite materials designed for targeted drug delivery. These materials maximize the therapeutic efficacy of an active ingredient while reducing its side effects. For instance, recent studies have ascertained the ability of such CNC-based nanocomposites to effectively deliver anticancer agents to sites within tumors while minimizing systemic toxic effects [99]. When considering stimulus-responsive systems, nanocellulosic functionality has allowed for the development of advanced drug delivery systems that will only release their therapeutic agents under specific pathophysiological conditions (pH or temperature changes). This is expected to increase the accuracy of treatments [100].

#### 3.1.2. Tissue Engineering

Nanocellulose is structurally similar to that of the extracellular matrix; therefore, it is the most suitable candidate among the various scaffolds for tissue engineering purposes. Among all the types, BNC has also been used for engineering skin, cartilage, and blood vessels because of its excellent mechanical properties, along with its biocompatibility.

In skin tissue engineering, BNC scaffolds meet the criteria for skin regeneration, being the ideal environment for the proliferation and differentiation of cell types. Studies have also revealed that BNC scaffolds sustain keratinocytes and fibroblasts, whose presence is vital in effective skin tissue engineering [101]. In addition, since BNC has high mechanical strength and flexibility, it is well-suited for cartilage tissue engineering. Different researchers have reported that chondrocytes have prolonged growth and could produce an extracellular matrix when used with BNC scaffolds for cartilage regeneration [98]. In the area of vascular grafts, the structural and biocompatibility properties of BNC have been manipulated for small vascular grafts. In vivo studies showed that BNC grafts integrate well with the host tissue and have a very low inflammatory response, indicating their great potential in vascular tissue engineering [102].

#### 3.1.3. Wound Healing

Nanocellulose-based dressings have demonstrated significant promise in wound healing applications due to their high absorption, permeability, and ability to provide an optimal moist environment for the wound healing process. In the case of moisture-retentive dressings, BNC dressings can provide a moist wound environment, which is basic for the fastest and most effective healing. Clinical studies have reported its effect on improving wound closure and lessening scarring by using BNC-based dressings [101]. In antimicrobial-infused dressings, nanocellulose dressings have already demonstrated the prevention of infections and are favored in fast healing when incorporated with antimicrobial agents such as silver nanoparticles. For instance, CNF-based dressings that incorporate silver nanoparticles have shown higher antibacterial activity and faster wound closure [98]. Nanocellulose dressings have shown their effectiveness in managing chronic wounds, for example, diabetic ulcers and others, through tissue regeneration while also reducing the days patients have to stay under treatment [100].

#### 3.1.4. Antimicrobial Applications

In antimicrobial coatings, nanocellulose can serve as a carrier for antimicrobial agents, forming coatings that can inhibit the colonization of bacteria on medical devices. It is found that bacterial colonization on surfaces is reduced by applying these nanocellulose composites containing metal nanoparticles or antimicrobial peptides [100]. In fact, antimicrobial agents that are embedded into a nanocellulose wound dressing are capable of preventing infections while accelerating the healing process. For instance, dressings based on CNFs loaded with silver nanoparticles exhibit good antibacterial activity and promote faster wound healing [103]. New types of implantable devices are made with an active polymer, nanocellulose, and are functionalized with antimicrobial agents to minimize the risk of infections after the surgery [74].

#### 3.1.5. Biosensing

The incorporation of CNCs and electrochemical sensors for glucose and other analytes offers a rapid and accurate measurement method. High sensitivity along with specificity laid the very foundation for effective biosensing applications [104,105]. In surface-enhanced Raman scattering (SERS) substrates, nanocellulose could be beneficial in preparing SERS substrates for biomolecule detection at even lower concentrations. Early disease diagnosis is generally made possible with this application [105]. It is highly believed that nanocellulose can have a great impact in the field of biomedicine, bestowing many properties, such as making it into a biodegradable, biocompatible, and mechanically versatile material. Through functionalizing and composite development, nanocellulose-based materials could prove to be efficient in improving therapeutic outcomes, inducing regeneration, and preventing infections by using alternative strategies.

### 3.2. Environmental Sustainability

Nanocellulose has started to gain a lot of interest because of its enhanced potential to become environmentally sustainable in almost every sector of the economy. There are several multidimensional functions of nanocellulose in promoting environmental sustainability, and the focus is on its applications in the water purification process, sustainable packaging, energy storage, and environmental remediation.

#### 3.2.1. Water Purification

Nanocellulose shows tremendous potential regarding its surface area and has the capacity to be functionalized to increase the affinity of a variety of pollutants. Thus, it can be used to remove heavy metals, dyes, and organic contamination from certain water sources, as demonstrated by several researchers. For example, it has been used to adsorb heavy metals, such as lead and cadmium, with very high removal efficiency. In addition, nanocellulose membranes function as a promising medium for the application of water filtration. Measured porosity and mechanical strength provide the opportunity for the effective removal of pathogens and particulates. Recently developed ultrafiltration membranes based entirely on nanocellulose could filter out bacteria and even viruses from contaminated water. The presence of antimicrobial agents in the nanocellulose architecture can avoid biofouling in filtration systems, resulting in an increase in the lifespan and performance of such systems. For instance, silver nanoparticle-imbedded nanocellulose membranes provided a prolonged antimicrobial effect and reduced contact with water-treatment microbial entities [106].

#### 3.2.2. Sustainable Packaging

Nanocellulose is intrinsically biodegradable and is therefore an eco-friendly option for packaging materials. It can be used to replace petroleum-based plastics, thereby reducing plastic pollution [107]. In addition, it can reinforce biopolymers, resulting in higher mechanical strength and durability. Studies indicated that the incorporation of nanocellulose in starch-based films increased their tensile strength and barrier properties, thereby enhancing their applicability for packaging. In the case of barrier properties, nanocellulose-based films are excellent barriers against oxygen and moisture, which is very important in food quality preservation. Coatings of nanocellulose can, according to the findings of some studies, significantly reduce oxygen permeability in biodegradable films, thereby prolonging the shelf life of perishable items [107,108,109].

#### 3.2.3. Energy Storage

As scaffolding for supercapacitor electrodes, cellulose provides a high surface area and high porosity that allow for efficient charge storage and transfer. Recent advancements have shown that cellulose/biopolymer composites deliver supercapacitors with high capacitance and stability. In lithium-resistant batteries, nanocellulose was also explored by researchers as a separator and electrolyte, thereby improving safety and performance. An example of this is a nanocellulose-based separator with better thermal stability and ionic conductivity, which would contribute to the safety and efficiency of batteries. Nanocellulose is flexible and biodegradable; therefore, it can be used in flexible and wearable electronics. Reports suggest its application is in the production of flexible supercapacitors and batteries, meeting the challenge of a growing need for green portable energy storage [110,111,112].

#### 3.2.4. Environmental Remediation

Functionalized nanocellulose aerogels have been synthesized to adsorb oil from water surfaces, acting as an eco-friendly solution for oil spill cleanup. These aerogels exhibit very high oil absorption capacity and can be reused after oil removal [113,114]. Heavy metal immobilization and other contaminants can be achieved using nanocellulose in soil, thus limiting their bioavailability and environmental impact. Several studies have confirmed that the use of nanocellulose amendments can enhance soil quality and improve vegetation growth in contaminated areas [115,116]. In air filtration, nanocellulose-based filters were developed to trap airborne pollutants, such as particulate matter and volatile organic compounds. It is known that the larger the surface area and porosity, the better they can improve indoor air quality [117,118].

Furthermore, CNCs and CNFs differ in morphology, crystallinity, and mechanical performance properties, which ultimately affect their suitability for use. A CNC itself is a useful colloidal suspension as its applications for performance are limited to mechanical strength applications, medical applications, and coatings. The properties of CNFs, by contrast, represent long, flexible, and tangled fibrils made of both amorphous and crystalline structures. A CNC is recognized and characterized to have a high aspect ratio; however, it is considered practically impossible to prepare a continuous network of CNCs. While a CNF has a high aspect ratio, it has the ability to trend towards network formation. This could serve as a more appropriate material to be used in applications such as hydrogels, rheology modifiers, flexible materials/substrates, water purification membranes, and inks for 3D printing, as well as a variety of conditions or uses that prioritize mechanical flexibility and film-forming capacity over crystalline properties.

### 3.3. Sensor Applications

#### 3.3.1. Gas Sensor

Currently, nanocellulose materials are being harnessed in establishing remarkable advances in fabricating gas sensors, fostering exceptional detection performance and environmental sustainability. Nanocellulose has demonstrated its potential in improving the performance and sensitivity of ammonia sensors. Electrochemical sensors derived from cellulose crystals/polyaniline (CNC/PANI) composites have showcased excellent proficiency in detection. It has achieved a rapid response time (less than 10 s) and a highly sensitive detection limit (10 ppm) [119].

The incorporation of titanium dioxide into the PANI/cellulose composite has resulted in advanced sensitivity, attributed to the formation of a P–N junction between PANI and TiO_2_. These PANI/TiO_2_/cellulose composites exhibited increasing resistance with ammonia exposure that decreased with desorption. Furthermore, the PANI/TiO_2_/cellulose composite is selectively sensitive to ammonia over other gases, highlighting its effective applicability [120].

#### 3.3.2. Chemical Sensor

Nanocellulose and its composites are commonly used in chemical detection owing to their unique characteristics, such as functional versatility and a large surface area. Nanofibrous PEI/BC-coated QCM sensors demonstrated outstanding detection performance for formaldehyde [121].

Catechol detection has been improved by using biosensors with laccase, CMC/CNF, and AgNP composites that demonstrate a superior selectivity, with a 1.64 µM detection limit. The detection limit has been further improved with Ag/ZnO with a lower limit of 0.205 µM [122]. Detection of toluene is aided with CNC-based composites with CB or RGO in rubber matrices [123]. Furthermore, nanocellulose-GQD hydrogels exhibited an ideal performance in detecting TCP (0.07 µg/mL), proving their potential for advanced hydrogel sensors [124].

#### 3.3.3. Enzyme Sensor

Nanocellulose-based sensors have emerged as effective platforms for enzyme detection. Human neutrophil elastase (HNE) is detected with high precision, aiding CNC-incorporated peptides. It has demonstrated a 10–20-fold improved sensitivity, outperforming conventional paper-based sensors. These sensors have detected HNE in wound fluids at 50 mU mL^−1^ by spraying a detectable dye upon the enzyme interaction [125]. Furthermore, the detection efficiency has been improved with fluorescent peptide-modified CNCs, achieving detection limits of 30 mU mL^−1^ for porcine pancreatic elastase and 50 mU mL^−1^ for HNE [126,127]. CNC films provide visual detection of xylanase and cellulase activity by changing color upon enzymatic hydrolysis, achieving a sensitivity up to 150 times higher than the other conventional methods. By changing the film architecture and CNC concentrations, selective and quantitative enzyme detection can be achieved [128].

#### 3.3.4. Ion Sensor

Nanocellulose-based fluorescent sensors have been developed to detect heavy metals in aqueous solutions. For instance, Fe^3+^ can be readily detected using so-called fluorescent probes; nonetheless, low selectivity and quenching effects have hindered their applicability in practical scenarios [129]. A recent study has demonstrated the potential of CNCs combined with pyrene derivatives in the detection of heavy metal ions, such as Pb^2+^, with high precision [130]. Another study involved developing ethyl cellulose-based nanofibers doped with CNINH (N′-(4-cyanobenzylidene) isonicotinohydrazide), enabling real-time environmental monitoring applications [131]. Nanocellulose materials have also been used to develop electrochemical sensors. A recent research study discovered the potential of incorporated nanofibers comprising pyromellitic dianhydride and dicyandiamide (PMDA/DCA) into electrochemical sensors for the efficient detection of Pb^2+^ ions [132], revealing the potential of designing nanocellulose-based composites to address specific pollutants.

Furthermore, nanocellulose composites have proven their ability to be utilized in tailoring electrochemical sensors that precisely detect nitrite ions. For instance, a CNC-integrated electrochemical sensor has evidenced successful detection of nitrite in water [133]. Additionally, CNC-based composites have proven their potential in detecting Hg^2+^ ions. In certain studies, efforts have been made to improve the affinity between nanocellulose and Hg^2+^ ions to achieve precise and efficient detection [134]. For the detection of radioactive contaminants such as uranium dioxide (UO_2_^2+^) in natural water samples, an innovative nanocellulose-based fluorescent sensor has been developed, contributing to the safer handling of radioactive materials [135].

#### 3.3.5. Glucose Sensor

Nanocellulose achieved high sensitivity for glucose detection. Polypyrrole (PPy)/CNC composites with immobilized glucose oxidase (GOx) exhibit an exceptional detection performance with a low detection limit (50 µM), a wide response range (1–20 mM), and selectivity against uric acid and ascorbic acid [136]. Correspondingly, cellulose nanofiber-based sensors with GOx coated onto electrodes showcased rapid response times. However, the sensitivity is hindered by the thickness of the coating, as thicker coatings weaken the diffusion of hydrogen peroxide [137].

**Figure 4:**
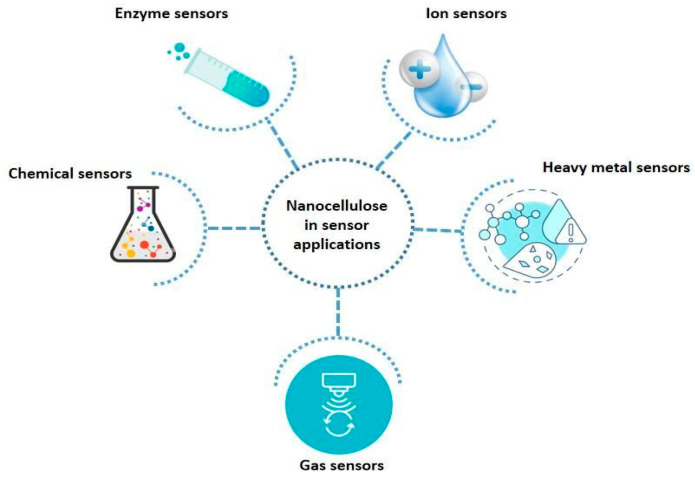
Nanocellulose in sensor applications.

In addition, nanocellulose plays a key role in developing non-enzymatic sensors. For instance, PDDA/CNC matrices with gold nanoparticles showcased improved sensitivity (62.8 µA mM^−1^ cm^−2^), enhanced selectivity, and a fast response (5 s), ensuring its proficiency in the subject [138]. Integration of CNCs with silver nanoparticles facilitated glucose detection, with a detection limit of 0.116 µM, through a visible color change, outperforming commercial kits [139]. Figure 4 further illustrates the overview of the sensor applications using nanocellulose.

### 3.4. Applications of Nanocellulose in Electronics

#### 3.4.1. Flexible Electronics

Owing to its unique characteristics, such as flexibility, transparency, exceptional mechanical properties, and coefficient of thermal expansion (CTE), nanocellulose can be considered an ideal material for substrates in flexible electronics.

At present, high-speed transistors have been manufactured by integrating silicon nano members (Si NMs) onto CNF films, achieving a frequency of 4.9 GHz, leading to sustainable electronics as they showcase biodegradability [140,141]. In addition, Si-based CMOS devices and gallium arsenide microwave devices on CNF substrates also exhibit outstanding performance, showcasing their potential for complete circuits [142]. Organic field-effect transistors (OFETs) made on transparent CNF nanopaper showcased their performance proficiency during blending, minimizing electrical shorting issues attributed to the strong bonding with carbon nanotubes (CNTs) [143]. Printed electronics, including flexible antennas and silver nanoparticle tracks on CNF substrates, have become ideal in their performance for RFID tags, sensors, and other applications compared to plastics, attributed to their favorable properties such as durability and lower resistance [144,145].

#### 3.4.2. Displays and Light-Emitting Diodes

CNFs have proven their potential as substrates for light-emitting diodes (LEDs) and displays, enabling transparent, flexible, and sustainable electronics. A recent study provides evidence for the successful fabrication of organic light-emitting diodes (OLEDs) on wood-based CNF composites. The fabrication comprises integrating acrylic resins upon acetylating CNFs and multiple deposition steps, including sputtering, spin-coating, and vacuum deposition, to create a functional OLED device [146,147]. In another study, OLEDs have been created on bacterial cellulose membranes via the mechanism of depositing indium tin oxide thin films at room temperature. This showcased outstanding performance, aiding future applications and achieving a luminance of up to 1200 cd/m^2^ [148]. In addition, Yagyu and his team fabricated light-emitting diodes on CNF substrates by curing the devices at 160 °C and printing silver nanoparticles, indicating the significance of CNF treatment in preventing discoloration and ensuring proper electrical conductivity [144].

#### 3.4.3. Optoelectronics

Another interesting application of cellulose nanomaterials is in liquid crystal displays (LCDs) and other electro-optical devices, given their potential to display liquid crystal behavior. The ability of CNCs to be aligned under an electric field makes them ideal for optical devices or displays [149]. The orientation of the CNC can be controlled by changing the electrical signal through the CNC suspensions; as such, the light transmittance can be controlled [150]. However, due to the limited amount of research in this domain, it is difficult to compare the efficiency of CNC on LCDs over the conventional materials for LCDs. Nonetheless, the optoelectrical effect is a promising characteristic of CNCs for favorable applications in the future.

#### 3.4.4. Energy Harvesting and Storage

Nanocellulose has been utilized in batteries, solar cells, energy-harvesting devices, and capacitors, effectively outperforming conventional materials. At present, photovoltaics has been modified with the incorporation of cellulose nanomaterials. A recent study has utilized CNF substrates coated with tin-doped indium oxide to innovate an organic solar cell, resulting in a lower power conversion efficiency than the control but showcasing the need for more improvements and investigations on the subject [151]. Another study utilized the optical haze (60%) and high transparency (96%) of CNF films, achieving a 10% improvement of power conversion efficiency via film lamination, resulting in an efficiency of 5.88% [152]. Zhou et al. utilized CNC substrates to fabricate a recyclable organic solar cell, resulting in an efficiency of 2.7% under a low energy requirement [153].

Nanocellulose plays a crucial role in battery applications, including separator membranes, flexible electrodes, and ion-conducting electrolytes. Flexible graphite–CNF electrodes have been fabricated with exceptional cycling performance [154]. In another study, CNF–CNT aerogels were utilized to fabricate flexible silicon-conductive nanopaper electrodes, achieving discharge capacities above 1200 mA h/g after 100 cycles, showcasing their performance proficiency compared to traditional graphite anodes [155]. Furthermore, CNF-based separators have shown their improved efficiency in fabricating safer and more effective Li-ion batteries owing to their favorable properties, such as improved thermal shrinkage, wettability, and ionic conductivity, outperforming commercial polypropylene/polyethylene separators [156,157].

### 3.5. Applications of Nanocellulose in Thin Films

Currently, polymer-integrated nanocellulose-based composite membranes have been used in ultrafiltration applications, showcasing their outstanding performance. The integrated polymers are mostly polyacrylonitrile (PAN) and polyethylene terephthalate (PET) [158]. A recent study has revealed the successful removal of positively charged dyes by utilizing CNC–chitosan membranes with nanoscale pores, where the mechanism of removal is aided by electrostatic attraction [159]. Another study has innovated hybrid membranes integrating palladium and graphene oxide nanoparticles, aiming at dye degradation, where the effort has been successful in achieving high efficiency in dye degradation and contaminant removal even under high-pressure conditions [160].

At present, nanocellulose-based materials are utilized widely for tissue bioscaffolds. This is supported by their unique favorable features, such as their biocompatibility and mechanical properties, which mimic natural tissues, assisting proliferation and cell attachment. Bacterial cellulose (BC) is known for its low cytotoxicity and high porosity [161,162]. CNC films fabricated with spin coating trigger an oriented cell morphology, achieving a feature height of 5–6 nm and facilitating skeletal muscle cell growth [163,164]. Human adipose-derived stem cell proliferation is supported by CNC electrospun nanofiber combinations owing to their favorable features, such as thermal stability and improved mechanical properties [165].

## 4. Challenges and Future Directions

### 4.1. Challenges

**Figure 5:**
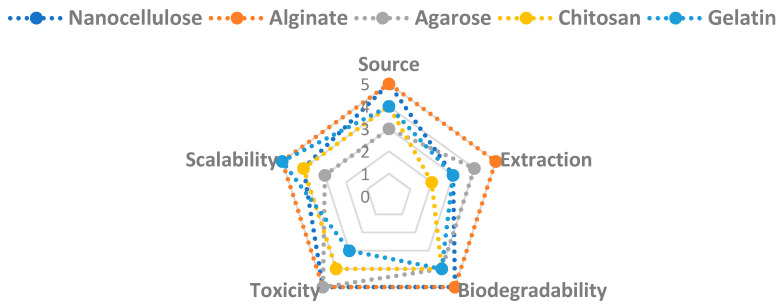
Comparison of the sustainability challenges of nanocellulose.

The versatility and sustainability of nanocellulose continue to draw attention, but a number of obstacles stand in the way of its large-scale manufacturing, functionalization, commercialization, and regulatory acceptance. The scalability, functionalization, regulatory barriers, and safety issues related to nanocellulose are examined in this chapter, along with suggestions for future approaches to deal with these issues. Furthermore, Figure 5 compares the sustainability challenges of nanocellulose with respect to naturally occurring biomaterials such as alginate, agarose, chitosan, and gelatin, focusing on challenging criteria such as the source, extraction procedure, biodegradability, toxicity, and scalability.

#### 4.1.1. Scalability Challenges

The high cost of raw materials, energy use, and specialized equipment are some of the primary barriers to increasing the production of nanocellulose. In order to produce CNCs, sulfuric acid hydrolysis requires careful handling and post-treatment to eliminate any remaining acid, which increases the operational complexity and expenses in chemical processes. Regarding the conversion of cellulose fibers into nanocellulose, techniques such as high-pressure homogenization and grinding require a large amount of energy, which makes them less cost-effective in mechanical processes. In addition, batch-to-batch variability results from the fact that the properties of nanocellulose depend upon the source of cellulose. Standardization is difficult since different plant fibers or biomass sources produce nanocellulose with different levels of mechanical characteristics, charge density, crystallinity, and yield [166].

Even if laboratory-scale manufacturing techniques are widely accepted, moving to industrial-scale production is still difficult. Optimizing large-scale reactors for enzymatic or acid hydrolysis processes is necessary for both financial viability and environmental sustainability. Slow microbial growth rates and the high cost of culture media hinder the scalability of biological processes, including the generation of bacterial nanocellulose [167]. Furthermore, concerns regarding the environmental sustainability of large-scale nanocellulose production are raised by the production of chemical waste, excessive water use, and substantial energy use [168].

#### 4.1.2. Challenges Due to the Presence of Lignin

Despite being a natural component of lignocellulosic biomass, residual lignin in nanocellulose can present challenges for applications requiring high purity. This is a critical consideration for the effective implementation of nanocellulose-based materials, especially on an industrial scale. For instance, studies have shown that residual lignin is cytotoxic to human cells, thus diminishing nanocellulose safety in medical applications such as wound dressing or tissue engineering [73,169]. It may, furthermore, interfere with cell growth and adhesion atop nanocellulose substances [170]. Additionally, lignin can interfere with sterilization methods, such as heat or radiation, discoloring the material chemically and thereby affecting its mechanical strength and performance [171]. In 3D printing for bioscaffolds, lignin’s peculiar dark color and heat resistance act against light or temperature control, which is required for the formation of precise structures [172].

Another aspect is that the use of nanocellulose in energy-related applications encounters challenges due to the residual lignin, particularly when sourcing partially purified biomass. Lignin can generate chemically active or insulating impurities, hindering the electrical and electrochemical properties of nanocellulose-based materials (e.g., catalytic interfaces and electrodes) and reducing their performance. Hence, the purity of nanocellulose must be a key concern when utilizing nanocellulose in energy-related devices such as solar cells, batteries, and supercapacitors. For example, ref. [173] demonstrated a 25% reduced capacitance of lignin-derived nanocellulose electrodes compared to lignin-free electrodes due to insulating residues generated by lignin, which led to conductive network disturbances. In addition, refs. [96,174] shows the structural irregularities resulting from lignin residues in nanocellulose-derived carbon components during carbonization. When it comes to the food industry, nanocellulose films are valued for their excellent oxygen barrier properties, especially under dry conditions. However, the presence of lignin can disrupt the dense hydrogen-bonded network, increasing permeability to gases and moisture [96,175]. Potential leaching of phenolic compounds from lignin residues could raise concerns about food safety compliance. In 2020, Yi Zhang and Maryam Naebe showed that although lignin provides good UV-shielding properties to the composite, it brought an undesirable dark color [176]. Therefore, effective delignification remains a critical step in nanocellulose production.

#### 4.1.3. Functionalization Challenges

The hydrophilic nature of nanocellulose restricts its compatibility with solvents and hydrophobic polymers. Surface functionalization is necessary to achieve uniform dispersion in hydrophobic matrices such as polypropylene or polyethylene, which increases complexity and expense. Functionalization techniques such as TEMPO oxidation and grafting frequently call for dangerous chemicals and several reaction stages. Even though harsh chemical treatments can break down cellulose chains, maintaining the mechanical and structural integrity of nanocellulose during functionalization requires careful balance. Although functionalization has advanced, it is still difficult to customize the characteristics of nanocellulose for particular uses. For example, improving hydrophobicity, electrical conductivity, or thermal stability frequently necessitates multi-step changes, which may restrict commercial viability [177].

#### 4.1.4. Regulatory Challenges

Inconsistencies in classification and regulation result from the different terms used to describe nanocellulose, including CNC, CNF, and BNC. Regulatory approval procedures are made more difficult by the absence of precise definitions and uniform characterization methodologies. The toxicity and environmental effects of nanocellulose are questioned due to its nanoscale nature. The lack of regulatory permission for broad usage is caused by the basic stage of comprehensive safety assessments, which include cytotoxicity, genotoxicity, and environmental destiny investigations. Strict regulatory frameworks require thorough testing of biocompatibility, biodegradability, and safety in applications including medicine delivery and food packaging. To guarantee their safety for human health, more assessments are required for chemical modifications of nanocellulose, such as carboxyl groups or sulfate esters. Moreover, the global commercialization of nanocellulose is complicated by the vast differences in regulatory requirements between nations. For instance, in the United States, the FDA might allow food contact products based on nanocellulose, while in the European Union, safety and environmental impact regulations might differ. Despite being generally regarded as harmless and friendly, nanocellulose’s tiny size presents possible health risks. Respiratory issues such as inhaling nanocellulose particles during production may cause lung inflammation, among other dangers comparable to those associated with other nanoparticles. According to studies, some cell lines may experience cytotoxic effects from high quantities of chemically modified nanocellulose [178,179,180].

Even though nanocellulose is widely regarded as environmentally friendly, if discharged in significant numbers, there are still worries about how it would behave in aquatic ecosystems, where it may interact with aquatic life or build up in sediments. We still do not fully understand the long-term impacts of nanocellulose exposure, especially in food or medical applications. Its breakdown routes, bioaccumulation, and interactions with biological systems require in-depth research.

### 4.2. Future Directions

Future developments should concentrate on creating economical and ecologically friendly extraction technologies, such as enzymatic or low-energy mechanical approaches to address scalability concerns. New functionalization techniques that preserve the structural integrity of nanocellulose while improving its characteristics include click chemistry and bio-inspired alterations. To make these techniques simpler for large-scale production, more research is required [170].

Multidisciplinary research on the toxicity, environmental effects, and long-term exposure concerns of nanocellulose will boost industry and regulatory trust and hasten its commercialization. Harmonizing national legislation and promoting international commercialization are two benefits of creating international standards for the manufacture, characterization, and use of nanocellulose. The growing use of nanocellulose in high-performance composites, water purification, and energy storage will raise its industrial demand and stimulate investment in scalable production techniques [181,182].

## 5. Conclusions

As a unique natural polymer from renewable biomass, nanocellulose is a new class of materials with excellent properties, such as high mechanical strength, large surface area, good biocompatibility, and biodegradability. In this review, we have summarized the extraction methods and applications of nanocellulose and highlighted the challenges and future directions.

Nanocellulose is prepared through mechanical, chemical, and biological approaches, which change the cellulose framework in specific ways to recover the nanocellulose in CNC, CNF, or BNC forms. All of the approaches discussed throughout this paper have strengths and weaknesses, highlighting the importance of continued research into cost-effective, scalable, and sustainable methods of production. Sustainable solvents such as ionic liquids (ILs) and deep eutectic solvents (DESs) have been studied as environmentally sustainable substitutes for traditional acid/base systems in nanocellulose extraction. ILs and DESs are less toxic than traditional acidic or alkaline systems, are recyclable, and provide lower overall environmental impact. Ionic liquids such as 1-ethyl-3-methylimidazolium acetate can disrupt the hydrogen bonding between cellulose fibers, allowing either the dissolution or disintegration of cellulose. DESs typically consist of H-bond donors and acceptors (choline chloride with urea), providing a low-cost, biodegradable, and environmentally friendly alternative to biomass fractioning that can provide pragmatic performance similar to ILs. The authors of [183] provide a comprehensive review of the potential for uses of IL and DESs, also demonstrating effective delignification and isolated cellulose from a variety of biomasses, with lower environmental impact and enhanced recyclability.

The potential of nanocellulose in many sectors, from packaging, electronics, and biomedicine to environmental remediation, is vast. There is still the need to overcome the issues of scalability, functionalization, and regulatory approvals, as well as safety challenges despite these advances. Further studies should be directed toward more efficient production, exploring novel functionalization approaches and developing sound regulatory systems and policies that support broader deployment. Nanocellulose remains an important component in making the transition away from fossil fuels and toward sustainable materials and a greener future.

## Figures and Tables

**Table 1:** Isolation methods and types of resultant nanocellulose using several natural sources.

Isolation Method	Source	Type of Nanocellulose	Dimensions and Yield of the Final Product	Ref.
Acid Hydrolysis (Acetic Acid)	Sisal fibers	CNF	27 ± 13 nm width 658 ± 290 nm length 60–70 wt% cellulose	
			10–15 wt% hemicellulose	[68,69]
			8–12 wt% lignin	
Acid Hydrolysis (Sulfuric Acid)	Tea stalk	CNC	4–8 nm width 49.87% yield	[69]
Acid Hydrolysis (Sulfuric Acid)	Garlic straw	CNC	6 nm diameter 480 nm length 80 aspect ratio 41% cellulose 18% hemicellulose 3.6% lignin	[70]
Acid Hydrolysis (Sulfuric Acid)	Cotton fibers	CNF	6–18 nm diameter 85–225 nm length 97.7 ± 2.2% cellulose 0.5 ± 0.4% hemicellulose 0.4 ± 0.1% lignin	[71]
Alkali Treatment + TEMPO Oxidation	Corn husks	CNF	8–10 nm width Aspect ratio > 300 66.5% cellulose 29.3% hemicellulose 2.6% lignin	[72]
Steam Explosion + Acid Hydrolysis	Pineapple leaf fibers	CNF	5–60 nm width 10.80 ± 0.50% moisture content 98.63 ± 0.54% cellulose 0.53 ± 0.03% hemicellulose 0.77 ± 0.44% lignin	[73]
Microwave Liquefaction + Chemical Treatment	Bamboo	CNF	2–30 nm diameter 83.67% cellulose 0.13% lignin	[74]
High Pressure defibrillation + Chemical Treatment	Hemp fibers	CNF	30–100 nm width Several micrometers in length 94% α-cellulose	[75]
Chemo-mechanical treatment	Wheat straw	CNF	10–80 nm diameter Few thousand nanometers in length 84.6 ± 4.41% cellulose 6.0 ± 1.1% hemicellulose 9.4 ± 0.8% lignin	[76]
Chemo-mechanical treatment	Soy hulls	CNF	20–520 nm diameter Few thousand nanometers in length	[76]
			94.0 ± 1.53% α-cellulose 3.5 ± 0.8% hemicellulose 2.5 ± 0.4% lignin	

**Table 2:** Properties of nanocellulose.

Properties of Nanocellulose	Examples	Numerical Ranges in Applications	Influencing Factors	Ref.
Mechanical Properties	Tensile Strength	2–6 GPa (CNFCs) 10 GPa (CNCs) 3–4 GPa (Microalgal CNFCs)	Crystallinity, Aspect ratio, Orientation	[86,87]
	Elastic Modulus	79–88 GPa (Bacterial CNFs) 56–220 (CNCs) 29–36 GPa (Wood CNFs)	Fibril entanglement, Matrix bonding	[86,87]
Thermal Properties	Thermal Degradation	355.56 ± 2.4 °C (BNCs 2 weeks of production) 368 °C (CNCs from Ramie fibers) 350 °C (CNFs from bleached wood pulp)	Crystallinity, Surface Modification	[88]
	Thermal Decomposition	-	Acid residues, Degree of oxidation	-
	Thermal Conductivity	~0.03–0.06 W/m·K Nano wood with naturally aligned nanocellulose	Density, Porosity	[89]
Optical properties	Transparency	Influence is negligible for B-CNF when at <10 wt%	Fibril diameter, Dispersion	[90]
	Haze	27.3–86.7% (Hazy transparent nanocellulose with 40 µm thickness)	Fibril uniformity, Surface roughness	[91]
	Transmittance	93–97% (when B-CNF is loaded up to 10 wt% in the composite films)	Film thickness, Dispersion	[90,91]
Structural Properties	Porosity	Mean pore diameter; 17.5 nm cellulose—FD 23.4 nm cellulose—SCD	Processing technique	[92]
	Morphology	CNCs: ~500 nm length; ~20 nm width) CNFs enlarged fibrils (15–100 nm width)	Extraction method	[45]
	Hydrophilicity and Functionalization	WCA 156° BNC membrane improved with (tridecafluoro-1,1,2,2-tetrahydrooctyl)-trichlorosilane	Surface hydroxyl/carboxyl groups	[93]
	Charge properties/Zeta potential	−40 to −60 mV (CNCs in aqua suspensions)	Sulfation, Oxidation, pH	[94]
Rheological Properties	Gelation	CNFs gel at ~1 wt% in water	Concentration, Fibril length	[95]
	Shear Thinning	Viscosity decreases with shear	Fibril entanglement	-
	Viscosity and Stability	-	Concentration, Surface chemistry	-
Environmental Properties	Biodegradability	Complete degradation within weeks/months	Origin, Treatment	-
	Sustainability	Vary upon renewable biomass	Biomass source	-
	Carbon Neutrality	-	Production method	-
Barrier properties	Gas Barrier Properties	-	Film density, Alignment	-
	Moisture Sensitivity	4.7 g mm/m^2^. day. kPa (Methyl cellulose-based 1 wt% CNC films)	Hydrophilicity, Treatment	[96]

## Data Availability

Data are contained within the article.

## Abbreviations

The following abbreviations are used in this manuscript:

CNC	Cellulose Nanocrystal
CNF	Cellulose Nanofiber
BNC	Bacterial Nanocellulose
NFC	Nano-fibrillated Cellulose
BC	Bacterial Cellulose
HPH	High-pressure Homogenization
TEM	Transmission Electron Microscope
XRD	X-ray Diffraction
TEMPO	2,2,6,6-tetramethylpiperidine-1-oxyl
xGnPs	Exfoliated Graphite nanoplatelets
NC	Nano Clay
PMDA/DCA	Pyromellitic Dianhydride and Dicyandiamide
GOx	Glucose Oxidase
CTE	Coefficient of Thermal Expansion
OFET	Organic Field-effect Transistors
CNT	Carbon Nanotubes
LED	Light-emitting Diodes
LCD	Liquid Crystal Displays
PAN	Polyacrylonitrile
PET	Polyethylene Terephthalate
IL	Ionic Liquid
DES	Deep Eutectic Solvent
